# When the Eagle Strikes the Carotid: A Case Report of Carotid Artery Dissection Secondary to Eagle Syndrome

**DOI:** 10.7759/cureus.112688

**Published:** 2026-07-15

**Authors:** Kiran Kishor Chandrasekar, Mariel Rogozinski, Angela Philips, Rubi Rodriguez Bobadilla, Kathleen Wiese

**Affiliations:** 1 Neurology, University of Illinois Chicago College of Medicine Peoria, Peoria, USA; 2 Neurology, Order of Saint Francis (OSF) Illinois Neurological Institute, Peoria, USA; 3 Emergency Medicine, University of Illinois Chicago College of Medicine Peoria, Peoria, USA

**Keywords:** carotid artery dissection, eagle syndrome, ischemic stroke, styloidectomy, styloid process

## Abstract

Eagle syndrome, characterized by elongation of the styloid process or calcification of the stylohyoid ligament, is a rare but clinically significant cause of carotid artery dissection and ischemic stroke that is frequently overlooked, particularly in younger patients without traditional cerebrovascular risk factors. We report the case of a 45-year-old right-handed man with no significant past medical history other than tobacco use who presented with acute-onset expressive aphasia and right hemiparesis following weight lifting. Initial neuroimaging revealed a left middle cerebral artery (MCA) territory infarct with occlusion of a distal M2 branch and left cervical internal carotid artery (ICA) occlusion suspicious for dissection. He was treated with intravenous tenecteplase and admitted to the neurological ICU. An extensive etiological workup, including echocardiography, cardiac rhythm monitoring, hypercoagulable studies, and cerebrospinal fluid analysis, was unremarkable. Computed tomography angiography (CTA) demonstrated bilateral elongation of the styloid processes, measuring approximately 5 cm each, with the left styloid process directly abutting the cervical ICA at the level of dissection, establishing Eagle syndrome as the underlying etiology. The patient subsequently underwent contralateral prophylactic styloidectomy following stabilization. This case highlights the importance of considering Eagle syndrome in the differential diagnosis of carotid artery dissection and ischemic stroke in young adults; careful review of neurovascular imaging, including assessment of styloid process morphology, is essential for timely diagnosis and prevention of recurrent cerebrovascular events.

## Introduction

Cervical artery dissection accounts for up to 20% of ischemic strokes in patients under 45 years of age and warrants consideration of rare structural etiologies [1, 2]. Eagle syndrome, first described by Watt W. Eagle in 1937, is defined by elongation of the styloid process (typically beyond 2.5-3.0 cm) or calcification of the stylohyoid ligament [3]. The condition classically manifests with cervicofacial or pharyngeal pain due to compression of adjacent cranial nerves and vascular structures. A less recognized but potentially devastating variant involves compression of the internal carotid artery (ICA) by the elongated styloid process, which may precipitate intimal injury, carotid dissection, and subsequent thromboembolic stroke [4]. This vascular subtype remains underdiagnosed, as the anatomical relationship between the styloid process and the carotid artery is not routinely assessed on standard neurovascular imaging.

Carotid artery dissection typically occurs spontaneously or in association with trauma, connective tissue disorders such as Ehlers-Danlos syndrome or fibromuscular dysplasia, or mechanical triggers including chiropractic manipulation and vigorous physical exertion [5]. Eagle syndrome represents a rare structural predisposition to dissection that warrants consideration in the appropriate clinical context.

We present the case of a 45-year-old man who developed a left ICA dissection with left middle cerebral artery (MCA) territory stroke, ultimately attributed to previously undiagnosed Eagle syndrome.

## Case presentation

Patient information and presenting concerns

A 45-year-old right-handed Caucasian man with a past medical history of smoking presented to the emergency department with acute-onset speech difficulty and right-sided motor deficits. Earlier that morning, he had been lifting weights at a gym when he experienced unusual visual changes, describing his vision as feeling “over-exposed.” He denied any associated headache, lightheadedness, dizziness, or blurred vision at that time.

Approximately two hours later, his wife observed that his speech had become significantly slurred. He subsequently developed profound weakness in his right upper extremity and mild weakness in his right lower extremity. He remained aware that his speech was impaired and that he could not articulate his thoughts. Emergency medical services were activated.

He denied any recent falls, head trauma, motor vehicle accidents, neck pain, or chiropractic manipulations. He reported that the weights he had been lifting were not unusually heavy. He was not taking any routine medications. Social history was notable for daily cigar smoking (5.25 standard cigarette pack-years or 1.5 cigar-specific pack-years). Family history was significant for an unspecified “bone disease” affecting multiple family members.

Clinical findings

On initial examination, the patient was afebrile with a blood pressure of 165/100 mmHg, heart rate of 80 beats per minute (regular), and oxygen saturation of 98% on room air. Neurological examination revealed an alert patient with profound expressive (Broca’s) aphasia, characterized by short, incomprehensible utterances with frequent paraphasic errors. Cranial nerve examination demonstrated a right upper motor neuron-type facial palsy. Motor examination revealed flaccid paralysis of the right upper extremity (Medical Research Council (MRC) grade 0/5) and paresis of the right lower extremity (MRC grade 3/5) [6]. Sensory examination showed diminished light touch and pinprick sensation in the right hemibody. Cerebellar testing was unremarkable. The National Institutes of Health Stroke Scale (NIHSS) score was 12 [7].

Diagnostic assessment

**Figure 1:**
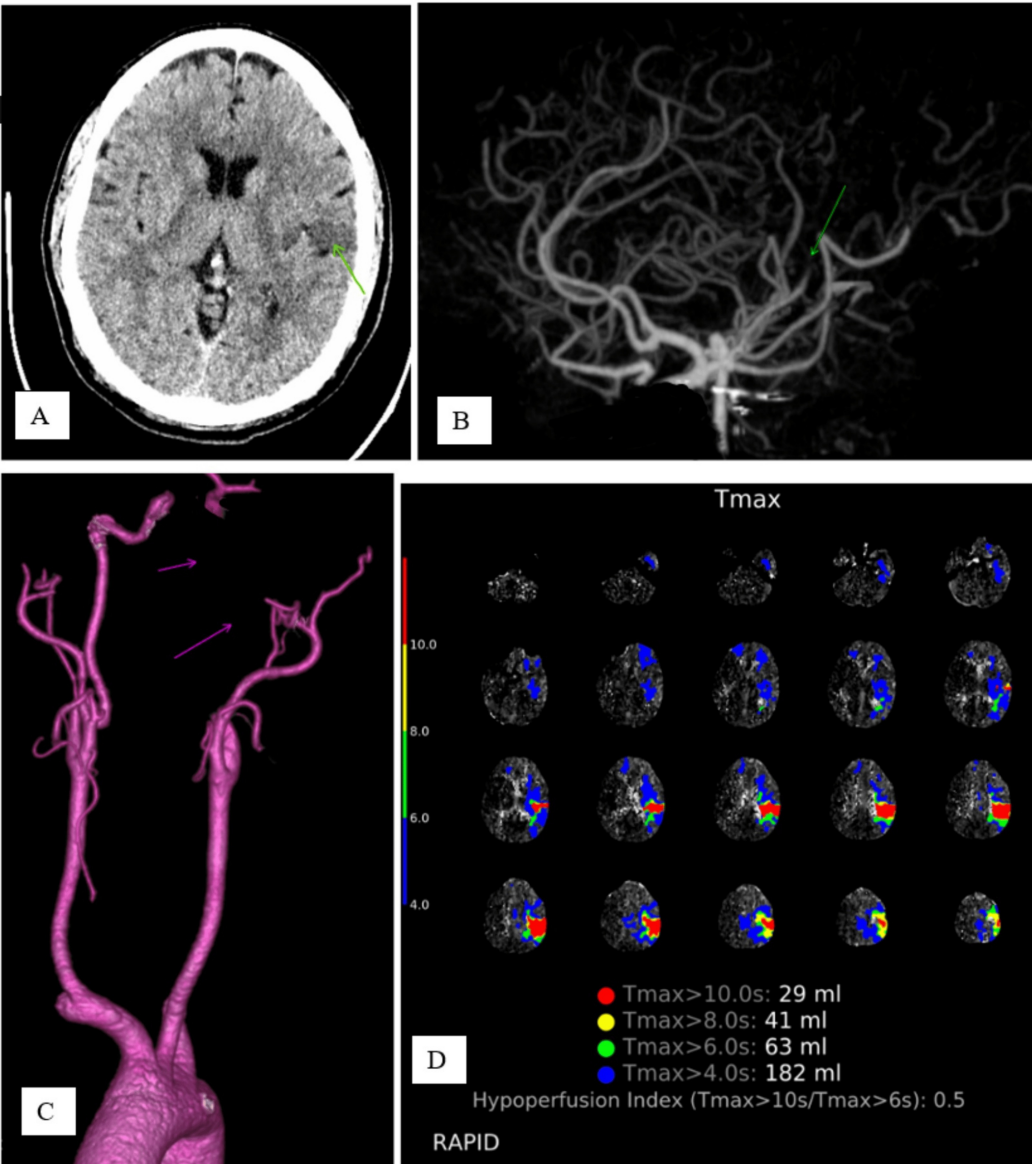
Acute neuroimaging on presentation (A) Non-contrast computed tomography (CT) of the head demonstrating subtle hypodensity with loss of the insular ribbon in the left insular cortex (arrow) and effacement of the adjacent frontal operculum. (B) Intracranial CT angiography maximum-intensity projection demonstrating abrupt cut-off of a distal M2 branch of the left middle cerebral artery (arrow), consistent with occlusion. (C) Three-dimensional volume-rendered CT angiography of the cervical vasculature demonstrating occlusion of the left cervical internal carotid artery (arrows), with reconstitution of flow at the petrous segment, consistent with dissection. (D) RAPID (Rapid Processing of Perfusion and Diffusion; RapidAI, Menlo Park, CA, USA) Tmax perfusion map demonstrating a left middle cerebral artery (MCA)-territory deficit (Tmax > 6 s = 63 mL) with a hypoperfusion index of 0.5. No ischemic core was identified on the corresponding cerebral blood flow (CBF) map (not shown), consistent with a favorable target-mismatch profile amenable to reperfusion therapy.

Emergent non-contrast computed tomography (CT) of the head demonstrated subtle early ischemic changes involving the left insular ribbon and frontal operculum, without evidence of hemorrhage (Figure 1A). CT angiography (CTA) of the head and neck revealed arterial compromise at two levels: intracranially, occlusion of a distal M2 branch of the left MCA (Figure 1B); and in the neck, occlusion of the left cervical ICA with reconstitution of flow at the petrous segment, findings suspicious for cervical carotid dissection (Figure 1C). CT perfusion, processed with RAPID (Rapid Processing of Perfusion and Diffusion; RapidAI, Menlo Park, CA, USA), showed a left MCA-territory deficit (Tmax >6 s = 63 mL) with a hypoperfusion index of 0.5; no ischemic core was identified, consistent with a favorable target-mismatch profile amenable to reperfusion therapy (Figure 1D).

**Figure 2:**
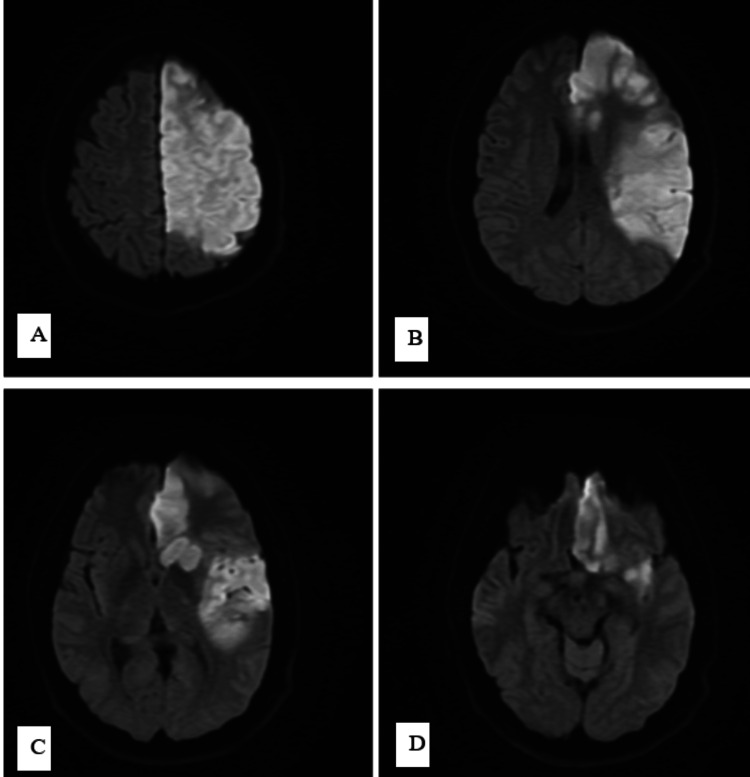
Acute infarct distribution on diffusion-weighted MRI (hospital day 4) Axial diffusion-weighted images at four levels demonstrate extensive left hemispheric acute infarction with restricted diffusion. The superior slices (A, B) show involvement of the medial/parasagittal frontal cortex, consistent with a left anterior cerebral artery (ACA)-territory component, while the more inferior slices (C, D) demonstrate left middle cerebral artery (MCA)-territory infarction involving the insula, operculum, and deep gray structures.

Serial imaging demonstrated infarct evolution, with diffusion-weighted MRI on hospital day 4 confirming extensive left hemispheric infarction involving both the MCA and anterior cerebral artery (ACA) territories (Figure 2). Because the ACA was patent bilaterally on the initial CT angiogram, the ACA-territory infarction is best attributed to the evolving nature of the dissection, delayed artery-to-artery embolism, as with the M2 occlusion, rather than to a fixed proximal occlusion present at onset.

Given the patient’s young age and absence of other traditional vascular risk factors, an extended etiological workup was pursued. Comprehensive testing, including transthoracic echocardiography, Holter monitoring, hypercoagulable panel (antiphospholipid antibodies, factor V Leiden, prothrombin G20210A mutation, proteins C and S), inflammatory markers, and cerebrospinal fluid analysis, was unremarkable.

Critically, review of the initial CTA demonstrated bilateral elongation of the styloid processes. The left and right styloid processes measured approximately 50 mm and 48 mm, respectively, along the curved long axis from the skull base to the tip on three-dimensional volume-rendered CTA reconstructions (normal <25-30 mm). 

**Figure 3:**
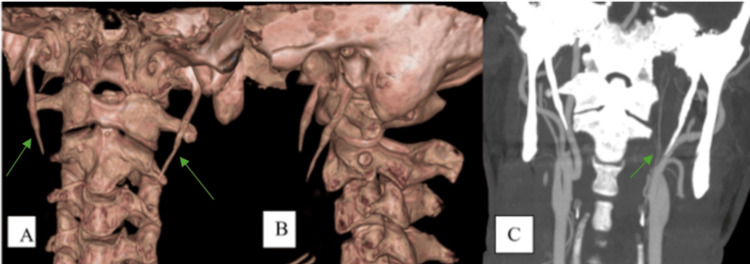
Eagle syndrome on CT angiography (CTA) with three-dimensional reconstruction (A) Anterior 3D volume-rendered CTA reconstruction demonstrating bilateral elongated styloid processes (~50 mm; normal <25-30 mm) projecting inferiorly from the skull base (arrows). (B) Oblique 3D volume-rendered reconstruction showing the elongated styloid processes in profile, coursing inferiorly toward the cervical carotid space. (C) Coronal CTA demonstrating the elongated styloid processes adjacent to the cervical internal carotid arteries (ICAs); the left ICA is non-opacified at the level of styloid contact (arrow), consistent with dissection and occlusion.

The tip of the left styloid process was in direct contact with the left cervical ICA at the precise level of the dissection. These findings, in the context of an otherwise negative etiological workup, established Eagle syndrome as the most likely cause of the carotid dissection and subsequent stroke (Figure 3).

The patient’s mother provided additional family history, reporting that many family members were unusually tall and appeared to “keep growing” throughout adulthood. She stated she was the shortest member of the family at 173 cm (5’8”), with siblings and other relatives being significantly taller. This history raised the possibility of a hereditary predisposition to styloid overgrowth, although formal genetic testing was not performed during this admission.

Therapeutic interventions

Based on imaging evidence of a distal M2 MCA occlusion with a favorable perfusion mismatch profile, the decision was made to proceed with intravenous thrombolysis using tenecteplase, as the last known well was two hours prior. Mechanical thrombectomy was considered but deferred, as instrumentation through a freshly dissected carotid artery carried a substantial risk of extending the dissection or causing further vessel injury.

The patient was admitted to the neurological ICU for continuous monitoring, permissive blood pressure management, and serial neuroimaging. Vascular surgery and interventional neuroradiology consultations determined that the patient was not a candidate for carotid endarterectomy or stenting in the acute-to-subacute phase given the severity of his neurological deficits and the high procedural risk associated with the dissected vessel.

He was initiated on aspirin 81 mg daily for secondary stroke prevention. Following neurological stabilization, a referral to otolaryngology was placed, and the patient subsequently underwent a right (contralateral) styloidectomy to mitigate the risk of future right ICA dissection, given the bilateral styloid elongation.

Follow-up and outcomes

The patient’s NIHSS score improved from 12 on presentation to 8 at discharge. The four-point NIHSS improvement (12 to 8) was attributable to partial recovery in facial weakness, right-sided sensory deficit, and right lower extremity strength, while expressive aphasia and dense right upper extremity weakness persisted. He was transferred to an acute inpatient rehabilitation facility with anticipated progression to outpatient therapy. The prophylactic right styloidectomy was performed without complications. Left-sided styloidectomy was not performed due to complete occlusion of the left internal carotid artery. 

Table 1 summarizes the chronological sequence of key clinical events during this episode of care.

**Table 1:** Timeline of key clinical events Chronological sequence of key clinical events during the index episode of care. AM: morning; h: hours; ED: emergency department; NIHSS: National Institutes of Health Stroke Scale; CT: computed tomography; CTA: computed tomography angiography; CTP: computed tomography perfusion; ICA: internal carotid artery; MCA: middle cerebral artery; ACA: anterior cerebral artery; IV: intravenous; ICU: intensive care unit; PFO: patent foramen ovale; TTE: transthoracic echocardiography; LP: lumbar puncture; CSF: cerebrospinal fluid.

Time Point	Event
Day 0 (AM)	Weightlifting at the gymnasium; onset of visual disturbance (“over-exposed” vision)
Day 0 (+2 h)	Acute-onset slurred speech, right upper extremity plegia, right lower extremity paresis; EMS activated
Day 0 (ED)	NIHSS 12; CT: early ischemic changes left insula/frontal operculum; CTA: left M2 occlusion, left cervical ICA occlusion (suspected dissection); CTP: penumbral mismatch. IV tenecteplase administered. Admitted to the neuro ICU.
Day 0 (+6 h)	Repeat CT: evolving left perirolandic and posterior insular infarct, no hemorrhagic conversion
Day 1	CT showed an evolving left MCA/ACA territory infarct with minimal mass effect and no hemorrhage. Aspirin 81 mg daily initiated.
Day 1–4	TTE: no thrombus, vegetation, or PFO. Holter: no atrial fibrillation. Hypercoagulable panel: negative. Inflammatory markers: normal. LP/CSF: no vasculitis or infection.
Day 4	MRI brain: stable infarct; CTA review: bilateral styloid process elongation (~5 cm), left styloid abutting cervical ICA at dissection level → Eagle syndrome diagnosed.
Day 7	Discharge to acute rehabilitation (NIHSS 8). Otolaryngology referral placed.
6 months post-discharge	Right (contralateral) prophylactic styloidectomy was performed without complications.

## Discussion

This case highlights several teaching points regarding Eagle syndrome as a cause of cervical artery dissection.

Diagnostic considerations

Eagle syndrome is estimated to be present radiographically in 4%-7% of the general population based on incidental findings of elongated styloid processes on imaging, though the vast majority of affected individuals remain asymptomatic [4]. The vascular subtype, involving compression or irritation of the ICA, is considerably rarer and accounts for an exceedingly small fraction of ischemic strokes. Consequently, the diagnosis is frequently delayed or missed entirely. In our patient, the diagnosis was established after a comprehensive negative workup for other stroke etiologies.

The temporal relationship between exercise and symptom onset in our patient is noteworthy. Weightlifting, particularly with associated Valsalva maneuvers and repetitive lateral neck flexion and shoulder shrugging, may generate dynamic mechanical stresses on the carotid artery at the point of contact with an elongated styloid process. This mechanism likely resulted in intimal injury, dissection, and subsequent artery-to-artery thromboembolism to the MCA territory [8].

The family history of unusual skeletal growth raises the possibility of a hereditary predisposition to styloid overgrowth. Familial Eagle syndrome has been reported, including in monozygotic twins [9], but the genetic basis remains poorly characterized.

Management considerations

The acute management of stroke secondary to Eagle syndrome follows standard acute ischemic stroke protocols. Our patient received intravenous tenecteplase based on favorable perfusion imaging, which demonstrated a significant penumbral mismatch. Mechanical thrombectomy was considered but appropriately deferred due to the risk of exacerbating the carotid dissection during catheter navigation through the injured vessel [10].

The optimal antithrombotic strategy following cervical artery dissection remains debated. The CADISS trial demonstrated no significant difference between antiplatelet therapy and anticoagulation for the prevention of recurrent stroke in cervical artery dissection [11]. In this case, aspirin monotherapy was preferred over anticoagulation, as the large completed infarct carried a heightened risk of hemorrhagic transformation.

The role and timing of styloidectomy in Eagle syndrome with cerebrovascular complications are not well defined, owing to the rarity of this presentation. In the acute phase, the priority must be stroke management and neurological stabilization. Surgical resection of the offending styloid process can be considered once the patient has stabilized, typically after three to six months. In our patient, contralateral (right) prophylactic styloidectomy was pursued to prevent a future dissection of the right ICA, given the bilateral styloid elongation. The ipsilateral (left) styloid was not addressed surgically, as the left ICA was already completely occluded.

Strengths and limitations

The strengths of this report include a thorough etiological workup that systematically excluded alternative stroke mechanisms, detailed serial neuroimaging, and clear demonstration of the anatomical relationship between the elongated styloid process and the carotid dissection. Limitations include the absence of formal genetic testing, which would have been informative given the suggestive family history. Additionally, long-term follow-up data regarding neurological recovery, vessel recanalization, and the durability of contralateral styloidectomy in preventing recurrent dissection are not yet available.

## Conclusions

Eagle syndrome should be considered in the differential diagnosis of carotid artery dissection and ischemic stroke, particularly in younger patients without a compelling conventional stroke mechanism. A high index of clinical suspicion, combined with careful assessment of styloid process morphology on CTA, is essential for timely diagnosis. Multidisciplinary management involving neurology, vascular surgery, and otolaryngology is critical for optimizing acute stroke outcomes and preventing recurrent cerebrovascular events through appropriately timed surgical intervention.
